# Bacterial dynamic of flue-cured tobacco leaf surface caused by change of environmental conditions

**DOI:** 10.3389/fmicb.2023.1280500

**Published:** 2023-11-28

**Authors:** Jie Ding, Kesu Wei, Xianchao Shang, Yuxue Sha, Liting Qin, Haozhen Li, Di Wang, Xiaohua Zhang, Shengjiang Wu, Delun Li, Feng Wang, Long Yang

**Affiliations:** ^1^Agricultural Big-Data Research Center and College of Plant Protection, Shandong Agricultural University, Taian, China; ^2^Guizhou Academy of Tobacco Science, Guiyang, China; ^3^Guizhou Provincial Academician Workstation of Microbiology and Health, Guiyang, China

**Keywords:** microbiological, dynamic change, flue-cured tobacco, environmental conditions, leaf surface

## Abstract

Microorganisms present on the surface of tobacco leaves play a significant role in shaping the composition of the tobacco microbial ecosystem, which undergoes continuous changes throughout the curing process. In the present study, a total of four distinct tobacco curing periods were selected for sampling, namely the fresh, yellowing, leaf-drying, and stem-drying stages. The bacterial 16S rRNA gene sequences of the collected samples were subsequently analyzed to identify operational taxonomic units (OTUs). The findings indicated that the complete dataset of leaf microbial samples was clustered, resulting in the identification of 1,783 operational taxonomic units (OTUs). Furthermore, the analysis of diversity revealed a pattern of initially increasing and subsequently decreasing community diversity. Redundancy Analysis (RDA) and weighted gene correlation networks for analysis (WGCNA) were employed in conjunction with environmental factors to assign OTUs to 22 modules for functional analysis. Additionally, a classification model utilizing the random forest algorithm was utilized to identify seven marker microorganisms (*Escherichia coli*, *Faecalibacterium prausnitzii*, *Faecalibacterium*, *Escherichia-Shigella*, Peptostreptococcaceae, Peptostreptococcales-Tissierellales, and Proteobacteria) that exhibited discriminative characteristics across different time periods. This study aimed to investigate the dynamic changes in the bacterial community throughout the curing process and their impact on the community’s function. Additionally, certain bacteria were identified as potential markers for detecting changes in the curing stage. These findings offer a novel opportunity to accurately regulate the curing environment, thereby enhancing the overall quality of tobacco leaf curing.

## Graphical Abstract

Microbial model of surface dynamic changes of flue-cured tobacco caused by environmental conditions.

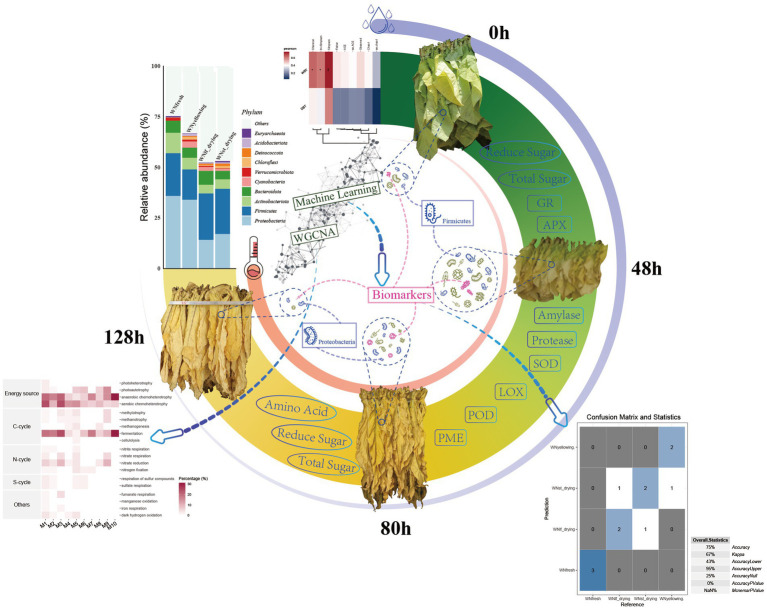

## 1 Introduction

Tobacco is an important cash crop that is widely grown around the world (Appau et al., 2020). After harvesting the mature cured tobacco, it undergoes a specific curing process to transform the fresh tobacco into dry tobacco (Li et al., 2021). Traditionally, the preparation of cured tobacco involved three distinct stages, as outlined by Zheng et al. (2017): the yellowing stage, the leaf-drying stage and the stem-drying stage. The composition of the foliar microbial community undergoes temporal changes in response to the curing house environment, leading to shifts in the dominant flora from one period to another (Glassman et al., 2018), and the biomarkers of the foliar microbial composition also vary from one period to the next (Zech et al., 2011). The microorganisms on the leaf surface of flue-cured tobacco undergo dynamic changes during the modulation process (Wu et al., 2021). During the flue-cured process, the temperature in the curing room undergoes incremental increases in stages, with each stage having a distinct duration for temperature retention, simultaneously, the humidity levels decrease in conjunction with the temperature increments (Wu et al., 2021). The fluctuating environmental conditions lead to concurrent alterations in the micro-ecological environment of the curing room. The physiological metabolism of the tobacco leaves is influenced by the environment, resulting in variations in respiration rate, surface metabolites, and inclusions. Additionally, this leads to dynamic changes in the fungal bacteria that parasitize the leaf surface (Dyakov et al., 2007). With the progression of the dominant flora and an augmentationin the abundance of both positively and negatively related species within the microbial community, discernible variations in community function and significant disparitiesin biomarkers emerged across differentstages. Microbial dynamics resulting from environmental changes have been extensively studied in fermented foods. Previous research has demonstrated similar community dynamics in white wine fermentation, highlighting the correlation between fermentation parameters and changes in microbial communities (Liberatore et al., 2010). Additionally, core microbial communities have been identified in soy sauce fermentation (Liu et al., 2021), and significant microbial succession has been observed in fermented foods like kimchi (Jung et al., 2014).

In this study, high-throughput sequencing technology was employed to investigate the microbial dynamics during the curing process using microbiomic methods. Specifically, foliar bacteria were amplified using 16 s amplicon sequencing technology, resulting in bacterial abundance tables. Statistical analysis was conducted on the obtained data to identify core functional microorganisms within the redundant microbial communities. Additionally, the study aimed to explore the changes in the diversity of foliar bacteria at different stages. This study aims to identify the microbial changes that occur during the curing stages. This study aims to elucidate the microbial succession pattern of cured tobacco and investigate the impact of environmental parameters on microbial succession.

## 2 Methods and material

### 2.1 Experiment material

Samples were taken from a tobacco station in Weining County, Guizhou Province, China. The tobacco variety was yun-87, the picking site was central, the picking time was mid-July 2020, and the tobacco was cured directly after picking. Samples were taken at 0, 72, 120 and 168 h, corresponding to the fresh tobacco, yellowing, leaf-drying and stem-drying, respectively. For each period, 5 leaves were selected and the base of the leaf and the main veins were cut off with sterilized scissors, wrapped in tin foil and snap frozen in liquid nitrogen (4 time points × 5 repetitions). Wet and dry bulb thermometer readings as an environmental factor in the cured room at the time of sampling (Supplementary Table S1).

### 2.2 DNA extraction and illumine mi-seq sequencing

The genomic DNA was extracted using the SDS method, followed by agarose gel electrophoresis to check the purity and concentration of the DNA, and the sample was diluted to 1 ng/μl using sterile water in a centrifuge tube. PCR is performed using diluted genomic DNA as template, and specific primers for amplification of V3-V4 variable region. The sequencing of the V3-V4 hypervariable region of 16S rRNA genes (primers: 5 -CCT ACG GRR BGC ASC AGK VRV GAA/T3 and 5 -GGA CTA CNV GGG TWT CTA ATC C-3). Efficient and high-fidelity enzymes were used for PCR with a Phusion® High-fidelity PCR Master Mix with GC Buffer (New England Biolabs, Ipswich, MA, USA). The PCR program was as follows: 98\u00B0C for 1 min, followed by 30 cycles of 98\u00B0C for 10 s, 50\u00B0C for 30 s and 72\u00B0C for 30 s, and a final extension at 72\u00B0C for 5 min. PCR products were detected by electrophoresis with 2% agarose gel. The samples were mixed in equal amounts according to the concentration of PCR products. After full mixing, PCR products were detected by 2% agarose gel electrophoresis. The target bands were recovered using gel recovery kits provided by Qiagen. Libraries were constructed using the TruSeq® DNA PCR-Free Sample Preparation Kit. The libraries were quantified by Qubit and Q-PCR and then sequenced using NovaSeq6000.

### 2.3 Data analysis

#### 2.3.1 Sequencing data processing

The reads of each sample were spliced using FLASH (V1.2.7) (Magoč and Salzberg, 2011) after truncating the Barcode and primer sequences from the downstream data, and the spliced sequences were The original Tags data (Raw Tags); the Raw Tags obtained by splicing, need to go through a strict filtering process to obtain high quality Tags data (Clean Tags). Referring to the Tags quality control process in Qiime (V1.9.1^1^) (Caporaso et al., 2010), the following operations were performed: (a) Tags interception: Raw Tags were intercepted from consecutive low quality values (default quality threshold <=19) base numbers The first low quality base site that reaches the set length (default length value is 3) is truncated; (b) Tags length filtering: Tags were intercepted to obtain the Tags dataset, and the Tags with consecutive high quality base lengths less than 75% of the Tags length were further filtered OTU. The Tags obtained after the above process need to be processed to remove the chimeric sequences, and the Tags sequence The Tags sequences were compared with the species annotation database by^2^ to detect the chimeric sequences and finally remove the chimeric sequences to obtain the final Effective Tags.

#### 2.3.2 OTU clustering and annotation

Using Uparse software (Uparse v7.0.1001^3^) (Edgar, 2013) to cluster all Effective Tags of all samples, the sequences were clustered by default with 97% agreement (Identity) into OTUs (Operational Taxonomic Units), while a representative sequence of OTUs will be selected, based on the principle of its algorithm, the sequence with the highest frequency of occurrence in OTUs is selected as the representative sequence of OTUs. Species annotation of OTUs sequences was analyzed using the Mothur method with the SSUrRNA database from SILVA138^4^ (setting a threshold of 0.8 to 1) (Quast et al., 2012) to obtain taxonomic information and at each taxonomic level separately: kingdom (boundary), phylum, class, order, family, genus, species, and the community composition of each sample. Rapid multiple sequence alignment was performed using MUSCLE (Version 3.8.31^5^) software to obtain phylogenetic relationships for all OTUs representative of the sequences.

#### 2.3.3 Diversity analysis

Unifrac distances were calculated and UPGMA sample clustering trees were constructed using Qiime software (Version 1.9.1). PCA, PCoA and NMDS plots were drawn using R software (Version 2.15.3). The Beta Diversity Index inter-group variance analysis was performed using R software, for parametric tests, *T*-test and wilcox test if there were only two groups, and Tukey test and wilcox test of the agricolae package if there were more than two groups. The ranking analysis was performed using the cca and rda functions from the vegan package. Based on species abundance, correlation coefficient values were calculated between genera and co-occurrence network analysis was performed using WGCNA (Langfelder and Horvath, 2008), igraph, pheatmap, dplyr and visualized using Gephi software (Bastian et al., 2009). Random Forest were using to build predictive models and find biomarkers markers.

## 3 Results

### 3.1 Changes in microbial abundance of cured tobacco at different stages

**Figure 1:**
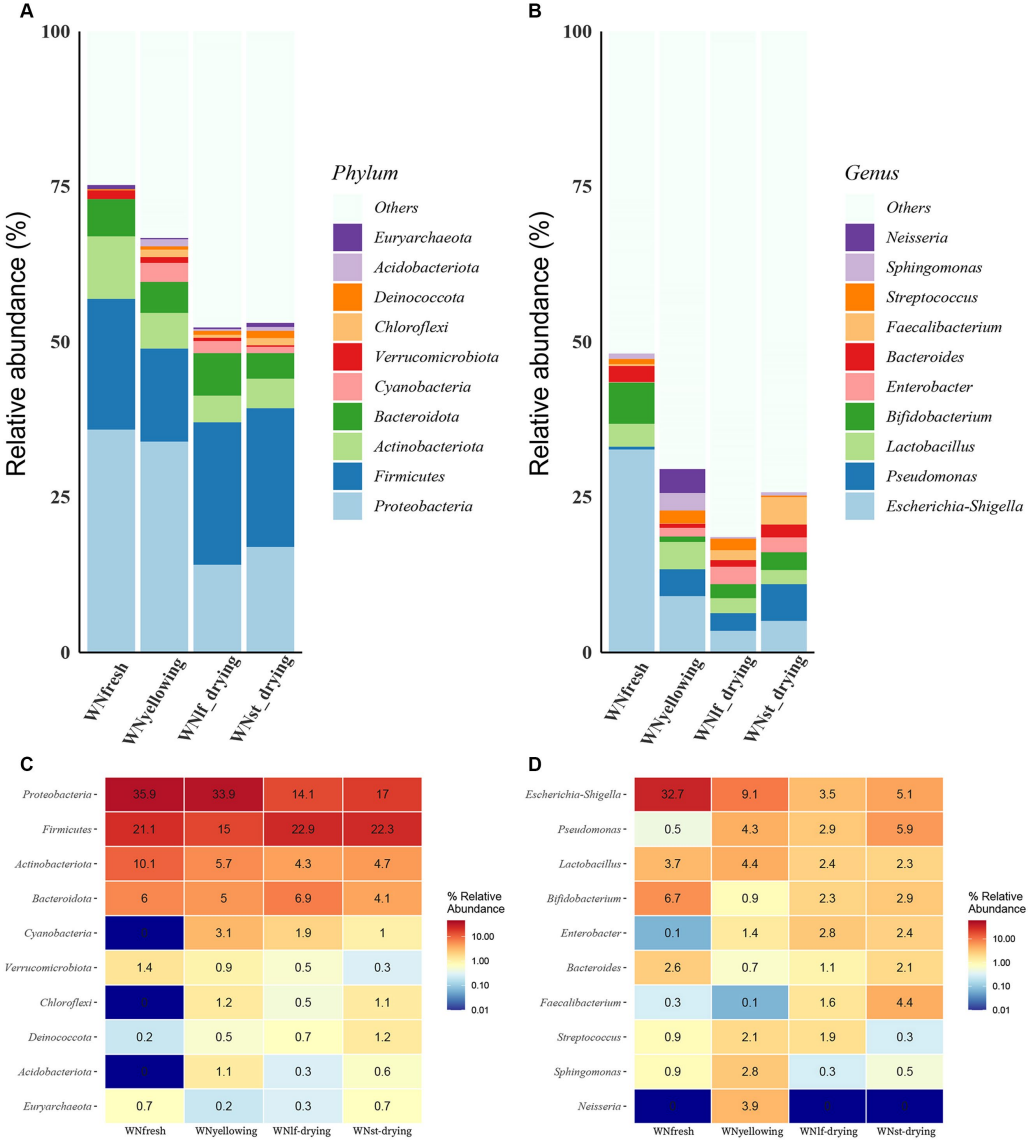
Comparison of bacterial communities at different curing stages. **(A)** Percent Stacked Column Chart of relative abundance of samples at the phylum level. **(B)** Percent Stacked Column Chart of relative abundance of samples at the genus level. Heat map of the top 10 relative abundances of samples at different taxonomic levels, with colors ranging from blue to red representing small to large relative abundances, and specific values of relative abundance indicated on each cell of the heat map. **(C)** Heat map of relative abundance at the phylum level. **(D)** Heat map of relative abundance at the genus level.

Microorganisms on the surface of tobacco leaves change during the curing process. A total of 2,993,443 tags were obtained in the whole microbial data set, and 1,783 OTU was obtained by clustering, annotated to 36 phyla, 77 classes, 154 orders, 217 families, 347 genera, and 404 species. Fresh tobacco leaves exhibit the highest bacterial count that can be classified at the phylum and genus levels. The phylum Proteobacteria account for 35.9 and 33.9% of the fresh and yellowing tobacco samples, and decrease significantly in the leaf-drying and stem-drying stages of mid to late curing, but the relative abundance of the Firmicutes increase, with 22.9 and 22.35% in the leaf-drying and stem-drying, respectively. The prevalence of Actinobacteriota is significantly higher in freshly harvested tobacco and gradually decreases throughout the curing process (Figures 1A,C). Bifidobacterium had the highest abundance in fresh tobacco leaves, with a significant decrease in abundance at the yellowing stage, followed by an increase in abundance at the leaf-drying stage and the stem-drying stage. Escherichia-Shigella had the highest abundance in fresh leaves, with an abundance percentage of 32.7%, which decreased significantly during the curing process. Enterobacter exhibited a low abundance of 0.1% in fresh leaves, but its abundance increased as the baking process advanced. It reached a maximum abundance of 2.8% during the leaf-drying stage, followed by a slight decrease in abundance during the stem-drying stage (Figures 1B,D).

### 3.2 Bacterial diversity and differences in bacterial communities at different curing stages

**Figure 2:**
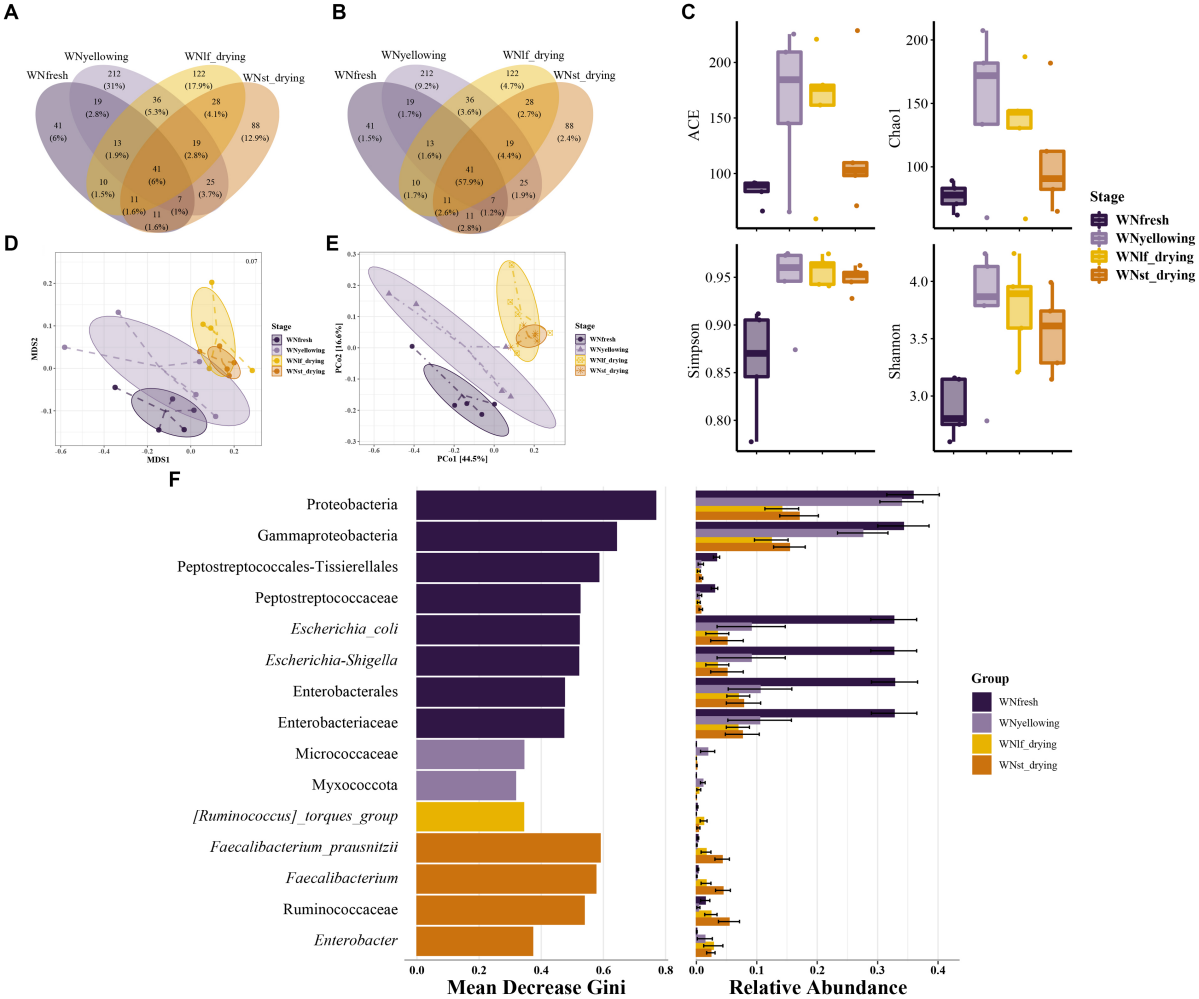
Bacterial diversity and flora differences at different curing stages bacterial Venn diagram of samples at different stages, one sample for each ellipse: **(A)** Numratio- Venn, the number on each unit represents the number of unique OTU of the unit, and the percentage represents the number percentage of all unique OTU of the unit; **(B)** Seqrate-venn, the number on each unit represents the number of unique OTU in the unit, and the percentage represents the percentage of representative sequences of all unique OTU in the unit **(C)** Boxplots were used to represent bacterial α diversity at different stages, and ACE index, Chao1 index, Simpson index and Shannon index were used to represent bacterial α diversity. The weighted distance matrix of bacterial communities among samples was calculated, and β diversity order analysis was performed based on the weighted distance matrix. Sample points in different curing stages were distinguished by different colors: **(D)** NMDS analysis with OTU level; **(E)** PCoA analysis of OTU level; **(F)** Random forest algorithm is used to predict biomarkers, MeanDecreaseGini is used to evaluate the importance of biomarkers, and the MeanDecreaseGini is ranked from high to low. The larger the value is, the greater the importance of the variable is, and at the same time the abundance of this biomarker in each group of samples is given on the right side of the bar chart of value, which is represented as a horizontal bar chart.

The results have revealed the core microbiota that is shared among the microorganisms present on the leaf during the yellowing stage. Additionally, the analysis has identified the operational taxonomic units (OTUs) that are unique to each sample. These findings have been summarized in a Venn diagram, as shown in Figure 2. The core dominant microbiota, represented by 41 common OTUs, accounted for only 6% of the total (Figure 2A). The total number of sequences accounted for 57.9% (Figure 2B), suggesting that the core dominant microbiota exhibits less variability. The yellowing stage exhibited the highest number of unique operational taxonomic units (OTU), with a total of 212 OTU, accounting for 31% of the total. However, these 212 OTU only represented 9.2% of the overall sequence count. The ACE and Chao1 indexes are frequently employed to assess the abundance of bacterial communities (Figure 2C). Both indexes consistently demonstrated a similar pattern, showing a significant increase in microorganism abundance during the yellowing phase, followed by a gradual decrease as the curing process advanced. The Simpson index and Shannon index exhibited a similar pattern in response to diversity, with lower values observed at the fresh leaf stage, followed by an increase during the yellowing stage, and subsequently a decrease. The principal coordinate analysis (PCoA) revealed that the PCo1 and PCo2 axes explained a significant portion of the community variation, accounting for 61.1% (Figure 2D). Additionally, the non-metric multidimensional scaling (NMDS) analysis demonstrated a low stress value of 0.07 (Figure 2E), indicating a strong ranking of the samples. Beta diversity analysis reveals that there is no distinct segregation between the leaf drying period and the stem drying period. Therefore, the NMDS analysis results are subjected to the Kruskal-Wallis rank sum test. The examination of the values reveals a notable disparity in community beta diversity between the two comparisons, namely yellowing - lfdrying and lfdrying - stdrying. In addition, there exists a notable distinction between the processes of yellowing and stdrying. The findings of this study suggest that the composition of the bacterial community undergoes changes during the modulation stage (Supplementary Figure S1). The application of the random forest algorithm in the field of machine learning enables the identification of various bacteria present on the leaf surface of tobacco at different stages of curing. This algorithm quantifies the influence of individual bacterial classification groups on the variability of observed values at each node of the classification tree. Additionally, the prioritization of the top 15 important and significant classification units is determined based on their average decrease in Gini value. Furthermore, the relative abundance of each classification group in each sample group is calculated, as depicted in Figure 2F. There exist distinct groups for classifying significant features in various sample groups during different time periods. Among these groups, fresh tobacco leaves exhibit the highest number of significant different feature classification groups. The phylum Proteobacteria holds the highest rank among feature classification groups in terms of significance. Additionally, within the phylum Proteobacteria, class Gammaproteobacteria is also considered to be of great importance. Family Peptostreptococcaceae, along with its taxonomic order Peptostreptococcales-Tissierellales, and family Enterobacteriaceae, along with its taxonomic order Enterobacterales, are of significant importance in the field. The prevalence of the two classification groups, Micrococcaceae and Myxococcota, is notably greater during the yellowing stage in comparison to the other stages. In the stage characterized by dry stems, the most prevalent and significant feature classification groups are Faecalibacterium and its member Faecalibacterium_prausnitzii. Additionally, Enterobacter is also identified as one of the significant feature classification groups in this stage.

### 3.3 The effect of cured room environment on leaf bacteria

**Figure 3:**
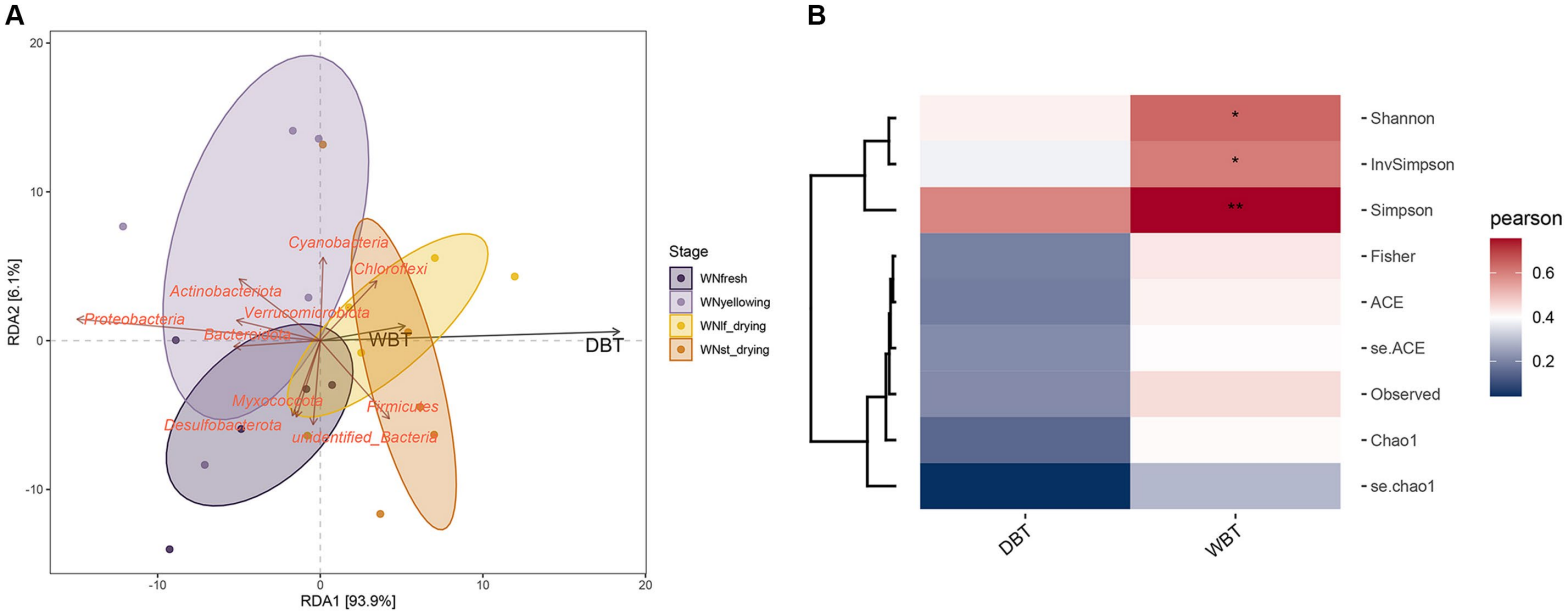
**(A)** Redundancy analysis of environmental factors, using different colors to distinguish different groups of samples. Environmental factors are represented by black arrows, and the arrows of feature vector and text are all red.**(B)** The correlation between the abundance of taxa and environmental variables is calculated, and the abundance of taxa is represented by α diversity index, *p< 0.05;**p< 0.01.

The diversity of microorganisms exhibited a strong correlation with the surrounding environment. The primary environmental indicator for assessing the condition of the cured room was the dry and wet bulb temperature (DBT/WBT), as it had a direct impact on the microbial abundance and diversity. The analysis of the correlation between dry and wet bulb temperature and the interleaf bacterial community at various stages was conducted using RDA (Figure 3A). The results indicated that DBT provided a more comprehensive explanation compared to WBT, and both variables exhibited a strong positive correlation with the RDA1 axis. Notably, the RDA1 axis accounted for a substantial proportion (93.9%) of the observed variation. Firmicutes and Chloroflexi exhibit a positive correlation with environmental factors, particularly dry and wet bulb temperature, thereby indicating their susceptibility to these conditions. DBT and WBT exhibited correlations with microbial diversity indices. Specifically, the wet bulb temperature (WBT) exhibited a significant correlation with the Shannon, Simpson, and InvSimpson indices, as depicted in Figure 3B. These indices are indicative of the microbial diversity in terms of α-diversity. Therefore, it can be inferred that the application of wet bulb temperature has a substantial impact on the diversity of phyllosphere bacteria throughout the curing process.

### 3.4 Bacterial co-occurrence network analysis and functional prediction

**Figure 4:**
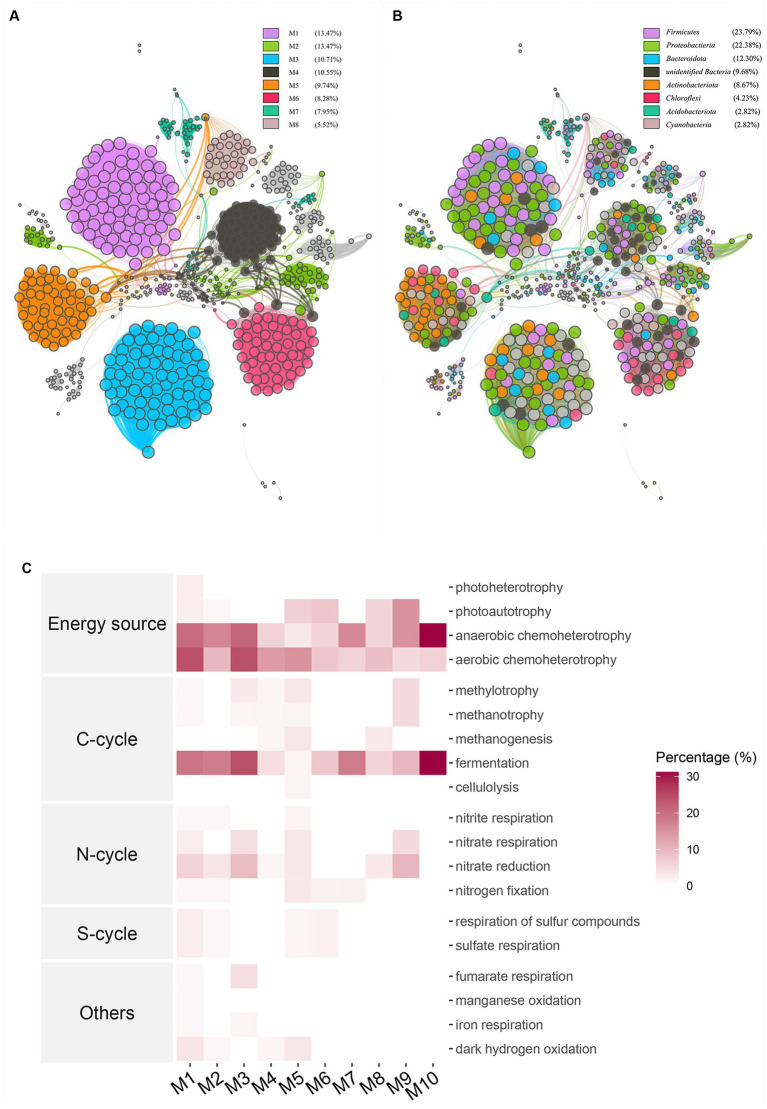
WGCNA co-occurrence network analysis showed potential interactions between all bacterial communities The lines connecting nodes (edges) represented the strength of the co-occurrence relationship color and the length of the edge represents the strength of the correlation in the unsigned co-expression network, the modules corresponded to OTUs with high absolute correlation. **(A)** Different colors were used to represent different modules, **(B)** Network map was colored according to taxa, **(C)** Functions of modules were located by using FARPROTAX database for comparison and the percentage of abundance of each module on each function was represented by heat map.

A weighted gene co-expression network analysis was performed on the complete 16S dataset in order to identify co-expressed modular colonies. The network analysis resulted in the identification of 614 nodes and 9,585 edges. These nodes were classified into 21 modules, with an average weighting of 29.581. The average network distance, or average path length, between all pairs of nodes was found to be 6.11 edges. Furthermore, the diameter of the network, representing the longest distance between any two nodes, was determined to be 18 edges. The obtained clustering coefficient was 0.886, indicating a high level of clustering within the network. Additionally, the modularity index was found to be 0.835, suggesting a significant degree of modular structure within the network. The eight modules with the highest proportion were chosen for colorization (Figure 4A). The phylum level taxa were color-coded in the same manner for different modules to enhance the visual examination of colony composition within the modules (Figure 4B). The largest module, M1, consists mainly of the phylum Firmicutes and the phylum Proteobacteria, while the two phyla with the greatest abundance, also stand out among the other modules, and phylum Actinobacteriota is highly represented in M3. Picrust2 was employed as a computational tool to predict community function and assess the function of the initial 10 modules within the colony. The enriched functions were primarily identified as Energy source, carbon cycle, nitrogen cycle, sulfur cycle, and others, as depicted in Figure 4C. The initial M1 modules primarily exhibited an enrichment in anaerobic chemoheterotrophy and aerobic chemoheterotrophy related functions in terms of energy source, as well as fermentation in the carbon cycle. On the other hand, module M3, while not directly connected to the other modules, showed a higher abundance of nitrate reduction and fumarate respiration compared to the other modules. Module M10 exhibited restricted connectivity, yet displayed higher abundance compared to other modules in both fermentation and anaerobic chemoheterotrophy. Functional predictions for the entire colony exhibited a higher degree of clustering within the broad categories of energy utilization and carbon cycle. This appearance may be directly linked to the subsequent fermentation process of aged tobacco leaves following curing.

### 3.5 Predicting curing stages using random forest classification model

**Figure 5:**
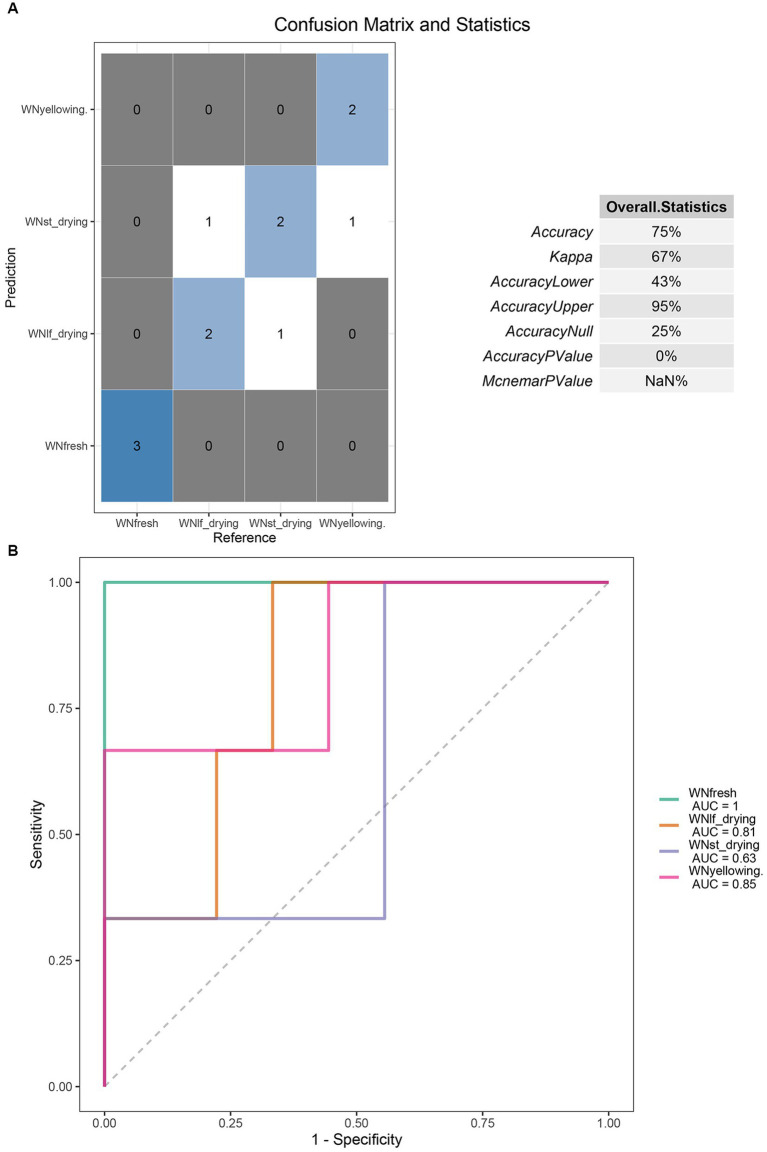
**(A)** Seven features OTU were selected to construct the random forest prediction model. Confusion matrix was used to represent the accuracy evaluation of the bacterial community prediction model in the curing stage. **(B)** The ROC curves of the four periods in the random forest prediction model were presented in the form of n rows and N columns. AUC is the area under the ROC curve.

Random Forest is a highly efficient machine learning algorithm that is based on the Decision Tree method. Decision Tree is a non-linear classifier that has the capability to capture intricate non-linear relationships among variables. By employing Random Forest analysis, it becomes feasible to discern the pivotal Operational Taxonomic Units (OTUs) that can effectively discriminate the dissimilarities between two sets of samples. Accurate identification of the degree of tobacco curing is essential for predicting tobacco curing stages. In this study, the entire dataset of leaf microorganisms was used as the training set, while 3/4 of the dataset was separated as the test set. A random forest model was constructed using this approach. The model included a total of 7 taxa (species *Escherichia coli*, *Faecalibacterium prausnitzii*, genus Faecalibacterium, Escherichia-Shigella, family Peptostreptococcaceae, order Peptostreptococcales-Tissierellales, phylum Proteobacteria) for prediction (Figure 5A). The prediction model demonstrates an accuracy rate of 75%. Among the fresh tobacco leaves, all three samples were correctly identified as fresh tobacco leaves. In the yellowing stage, two samples were accurately classified as yellowing tobacco leaves, while one sample was identified as being in the stem drying stage. In the leaf-drying stage, two samples were accurately predicted, while one sample was incorrectly classified as being in the drying stage. In the stem-drying stage, two samples were accurately predicted, while one sample was incorrectly classified as being in the coloring stage. These prediction results demonstrate that the model possesses the capability to accurately identify the temporal variations in the tobacco drying procedure. Additionally, the kappa coefficient of 0.67 further confirms the accuracy of this prediction model. The kappa coefficient of 0.67 indicates the level of accuracy exhibited by the model. The accurate results in the prediction model are distributed along the diagonal line, indicating the precision of the model’s predictions. This is crucial for determining the drying stage. The receiver operating characteristic (ROC) curve demonstrates a high level of confidence, as indicated by a false positive rate of 0 and an area under the curve (AUC) value of 1 for fresh tobacco. Additionally, the AUC values for the yellowing and color setting stages are 0.81 and 0.63, respectively (Figure 5B).

## 4 Discussion

Previous research on tobacco microbes has primarily focused on the examination of endophytic microorganisms and rhizosphere microorganisms during the growth stage of tobacco (Chen et al., 2015). Other studies have focused on microbial changes during the post-curing aging stage and the fermentation of cigars (Liu et al., 2021). Additionally, there have been studies that specifically investigate microbial changes during the post-curing aging phase and cigar fermentation (Liu et al., 2021). Nevertheless, there is a scarcity of research on microorganisms during the curing process, and the impact of environmental factors on microbial transformations during this procedure remains ambiguous. In this study, the utilization of microorganisms was employed to systematically investigate the alterations in the bacterial community that take place during the curing process.

The microbial community demonstrates unique attributes and experiences fluctuating transformations at various stages during the curing process, which can be discerned by examining operational taxonomic units (OTUs). The curing process can be considered as a fermentation process characterized by high temperatures and short durations, which are influenced by factors such as temperature and humidity. Temperature and humidity are widely recognized as the primary factors influencing microbial alterations. It has been discovered that alterations in environmental factors have an impact on the attributes of tobacco at the macro level, as well as on microbial changes at the micro level (Brockett et al., 2012). In the current investigation, the prevailing phyla at the phylum level were determined to be Firmicutes and Proteobacteria. Nevertheless, a notable decline in the relative abundance of Proteobacteria was observed during the leaf-drying stage and the stem-drying stage under elevated temperatures. Conversely, there was an observed increase in the relative abundance of the Proteobacteria phyla during these stages. This finding implies that the dominant taxa within the Proteobacteria experienced a more pronounced impact as a result of elevated temperatures (Mateos-Rivera et al., 2016). On the contrary, the observed rise in the abundance of the Firmicutes phylum suggests that it harbored a larger population of organisms that exhibited tolerance to elevated temperatures. The diversity of bacteria exhibited significant changes during the curing process. Alpha diversity analysis demonstrated that the yellowing stage exhibited greater species richness and diversity in comparison to the fresh tobacco stage. It has been postulated that the initial rise in temperature and humidity during the curing process creates an optimal environment for the growth of bacterial flora (Li et al., 2020). This proliferation, however, diminishes after the yellowing stage due to the sustained increase in temperature, which inactivates certain bacteria (Feller and Gerday, 2003). Additionally, the respiration of tobacco leaves consumes a substantial amount of oxygen, leading to a decrease in the activity and abundance of aerobic bacteria. Conversely, this decrease in oxygen availability results in an increase in the number and diversity of anaerobic bacteria (André et al., 2021). Notably, the anaerobic bacterium Faecalibacterium_prausnitzii, which is highly sensitive to oxygenation, exhibited a significant up-regulation in abundance during the drying period (He et al., 2021). This finding was determined through analysis of variance using the Random Forest algorithm. The presence of Faecalibacterium_prausnitzii, an anaerobic bacterium highly susceptible to oxygen levels, was notably increased during the drying stage. Additionally, Ruminococcus, another genus that thrived during the drying process, has been known to break down cellulose and hemicellulose, potentially contributing to the enhanced flexibility of tobacco (Ze et al., 2012). Beta diversity analysis reveals that the microbial community on the leaf surface undergoes sequential changes throughout the curing process. The community structures observed during the leaf drying and the stem drying period exhibit a partial overlap, suggesting a tendency for microbial communities to stabilize in the later stages. Significant differences, as determined by NMDS analysis, are still evident in the samples collected from these two periods. This phenomenon can potentially be attributed to variations in the composition and population size of fundamental microbial communities.

In the present study, an analysis was conducted to examine the association between bacterial community α-diversity and environmental factors. We conducted a study to investigate the impact of dry and wet bulb temperatures on diversity in the curing room, considering its relatively enclosed environment and the significant influence of environmental factors. Our RDA analyses revealed a positive correlation between both environmental factors and the abundance of Firmicutes and Chloroflexi, two taxa that play a crucial role in the symbiotic network. The functional enrichment of the bacterial community is predominantly focused on the carbon and energy cycles (Patel et al., 2021). Upon introducing fresh tobacco into the curing room, the temperature increase led to a notable rise in the respiration rate of the tobacco. This rise in temperature also corresponded to a decrease in the initial bacterial energy source, primarily composed of starchy substances. Additionally, during this period, aldehydes, ketones, and other primitive flavor substances began to accumulate. In the later stages of curing, these substances gradually transformed into proteins and other nitrogen-containing compounds. The presence of flavor substances in tobacco is closely related to the overall function of bacteria. Notably, the M3 and M9 modules of the nitrogen cycle were significantly enriched in functional modules. This enrichment may be attributed to the decomposition of nitrogenous compounds on the surface of tobacco leaves and the subsequent neutralization of tobacco flavor after curing.

Microorganisms play a crucial role in determining the quality of leaves during the curing process. It is worth noting that both rapid and gradual heating, as well as variations in humidity, can potentially impact the overall quality of tobacco (Chen et al., 2021). In the context of tobacco production, it is important to note that the quality of tobacco leaves can vary across different production seasons, origins, and parts of the plant (Tang et al., 2020). As a result, the standard curing curve used in traditional curing processes may not be suitable for all tobacco leaves. In this study, a random forest prediction model was developed to forecast the curing period. Seven feature OTUs were carefully chosen to enhance the accuracy of the prediction, resulting in a 75% accuracy rate. This model offers the potential to improve the control of the curing process by allowing for the evaluation of specific periods and enabling more precise adjustments of temperature and humidity based on the microorganisms and tobacco conditions during production (Tang et al., 2020). It is important to highlight that the training dataset used for the prediction model in this study was not adequately comprehensive. In future tobacco production, it is recommended to collect more robust data for large-scale microbiological data collection. Additionally, gathering more physiological and biochemical indicators of tobacco quality would be beneficial in order to investigate the correlation between bacterial changes and tobacco curing (Yan et al., 2022). Furthermore, it is worth noting that this study solely focused on the collection of 16 s rDNA data. It is important to acknowledge that the fungi present on the leaves play a significant role in actual production (Chen et al., 2020). Therefore, exploring the interleaf fungi also presents a wide range of research opportunities.

## 5 Conclusion

In this study, high throughput sequencing technology was used to analyze and quantify the bacterial communities on leaf surface in different curing periods. The results showed that the leaf surface had abundant bacterial community structure, and there were dynamic changes in the process of curing, and the bacterial community on the leaf surface had high modular unity in function, resulting in a series of macro changes in the quality of tobacco leaves. The selection of some taxonomic groups as predictive factors provided a new perspective for the precise control of tobacco curing. In future studies, more environmental factors should be integrated to better characterize the distribution and function of flue-cured tobacco surface community, simulate microbial changes on flue-cured tobacco leaf surface, and realize precise regulation guided by microbial changes.

## Data availability statement

The data presented in the study are deposited in the NCBI repository, accession number PRJNA1008596.

## Author contributions

JD: Conceptualization, Methodology, Software, Visualization, Writing – original draft. KW: Data curation, Funding acquisition, Resources, Writing – review & editing. XS: Conceptualization, Investigation, Software, Writing – review & editing. YS: Methodology, Software, Writing – original draft. LQ: Formal analysis, Methodology, Writing – original draft, Writing – review & editing. HL: Methodology, Software, Writing – original draft. DW: Methodology, Supervision, Validation, Visualization, Writing – original draft. XZ: Data curation, Software, Writing – original draft. SW: Data curation, Writing – review & editing. DL: Data curation, Writing – review & editing. FW: Data curation, Writing – review & editing. LY: Data curation, Funding acquisition, Methodology, Project administration, Resources, Supervision, Writing – review & editing.

## Footnotes

^1^ http://qiime.org/scripts/split_libraries_fastq.html

^2^ https://github.com/torognes/vsearch/

^3^ http://www.drive5.com/uparse/

^4^ http://www.arb-silva.de/

^5^ http://www.drive5.com/muscle/
