# Supplementary material for: Bacterial dynamic of flue-cured tobacco leaf surface caused by change of environmental conditions
Source: Front Microbiol. 2023 Nov 28;14:1280500. doi: 10.3389/fmicb.2023.1280500 (PMC10713716; doi:10.3389/fmicb.2023.1280500)

Table S1. Dry bulb and wet bulb temperatures in the cured room at the time of sampling.

	DBT (℃)	WBT (℃)
Fresh	30.0	/
Yellowing stage	42.0	37.0
Leaf-drying stage	54.0	39.0
Stem-drying stage	68.0	41.0

Fig. S1. Between-group NMDS distance significance test (P＜ 0.05)

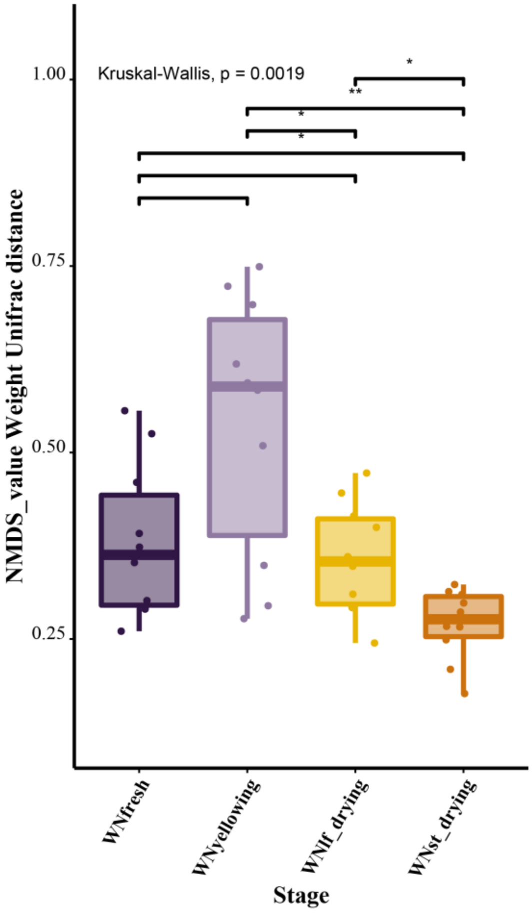

Supplement: Supplementary file 1 [file Data_Sheet_1.PDF]
